# Emergency Department Visits and Hospitalizations for Buprenorphine Ingestion by Children — United States, 2010–2011

**Published:** 2013-01-25

**Authors:** Maribeth Lovegrove, Lee M. Hampton, Daniel S. Budnitz, Andrew I. Geller

**Affiliations:** Div of Healthcare Quality Promotion, National Center for Emerging Zoonotic and Infectious Diseases; EIS Officer, CDC

Buprenorphine (Subutex) and buprenorphine/naloxone (Suboxone) received Food and Drug Administration approval in 2002 for the treatment of opioid dependence. Introduction of these drugs expanded the availability of opioid-dependence treatment options to reduce the morbidity and mortality associated with opioid abuse, and buprenorphine has become an increasingly prescribed component of office-based treatment. However, unsupervised ingestion of buprenorphine-containing products by children is a growing concern (1).

During 2010–2011, the National Electronic Injury Surveillance System — Cooperative Adverse Drug Event Surveillance project (2) identified 68 cases involving buprenorphine product ingestions (out of 226 cases of opioid product ingestions) by children aged <6 years.* Based on these cases, CDC estimates that, during 2010–2011, an average of 1,499 (95% confidence interval [CI] = 905–2,092) children aged <6 years were evaluated each year in U.S. emergency departments (EDs) for buprenorphine-product ingestions; in contrast, zero cases were reported in 2004. Nearly all (95.8%) of the ED visits involved buprenorphine/naloxone. As is typical of unsupervised pediatric exposures to other medications, most ED visits involved boys (59.5%) and children aged 1 and 2 years (76.8%). Because of buprenorphine’s long half-life and risk for respiratory depression, 58.4% of ingestion-related ED visits required hospitalization.†

Buprenorphine products were involved in disproportionate numbers of unsupervised ingestions by children aged <6 years, compared with other prescription drugs. Buprenorphine products were involved in 29.8% (CI = 20.1%–39.5%) of ED visits and 59.5% (CI = 38.9%–80.2%) of emergent hospitalizations for opioid product ingestions.§ Ingestion of buprenorphine/naloxone caused 9.5% of emergent hospitalizations for drug ingestion by children aged <6 years, a greater proportion than any other single medication, even though in 2009 buprenorphine products amounted to only 2.2% of all retail opioid prescriptions and 0.16% of all retail prescriptions (3).

Ingestions of buprenorphine-containing products by children are serious and have increased rapidly nationally. Fatalities have been reported in children after unsupervised ingestions of single doses (4). Innovative injury prevention approaches beyond current child-resistant bottles and safety warnings might be required to safeguard children while preserving availability of medications such as this important addiction treatment option for adults. Providers should remind patients to keep buprenorphine-containing products in child-resistant packaging, stored up, away, and out of the sight of children. Patients should post the Poison Help telephone number (1-800-222-1222) in their homes, and telephone 911 in the event of emergencies.

## Footnotes

* A case was defined as an emergency department (ED) visit at one of the participating NEISS-CADES hospitals during 2010–2011 for assessment and treatment for a buprenorphine product ingestion by a child aged <6 years, where the child accessed the medication on his/her own, without adult permission or oversight (unsupervised ingestion).

† Hospitalizations included ED visits that resulted in admission to a hospital or transfer to another hospital.

§ Excluding opioid-containing antitussive formulations.
